# Hygrometric Conditions of the Atmosphere

**Published:** 1854-07

**Authors:** S. B. Hunt


ART.—V.—*Hygrometric conditions of the Atmosphere.* Communicated to the Buffalo Medical Association, by S. B. HUNT, M. D.

The attention of etiologists has long been directed to the condition of the atmosphere, as a most likely source from whence may arise diseases of various types. It has become an axiom, that a pure air is the great necessity for the preservation of health. Accordingly, the research of those who would seek out, and, if possible, alleviate the causes of disease, has been especially directed to aerial conditions. Certain influences have been recognized, and their phenomena pointed out. The exhalations of marshes have received the name of malaria, and a certain class of diseases, whose causation has been assigned to them, have been named malarious diseases. Again, other causes of atmospherical impurity, among which may be enumerated the filth of cities and intramural interments, have been investigated, and found to exert most deleterious effects upon the general health.

But it is an unfortunate truth that these conditions are not appreciable by chemical analysis. What they are, and what shall best prevent them, are questions which we have no tests delicate enough to solve. That they do exist is evident by the slow, but sure, chemical reactions they eventually produce upon the human frame, as well as by those tests with which nature has gifted us, the organs of special sense. An impurity of the air not appreciable by the most careful manipulations of the chemist, is frequently very easily recognized by the olfactory organ, and most men who have lived in malarial districts are familiar with the nauseous taste of a miasmatic fog. Leaving for a time the consideration of this branch of epidemiology, let us turn to those conditions which are appreciable, and see if we can there find any causes of disease.

There is, of course, some normal standard of atmospheric purity—some equal balance of all its elements—which shall be best adapted to the purposes of the human organism. Could we fix upon this, and then assign to all variations from it their due degree of importance, we should, by explaining the origin, go far to prevent the presence of disease.

We are told by chemists that the atmosphere is a mechanical mixture of two gases, oxygen, and nitrogen; and that it contains a proportion of carbonic acid, and a trace of ammonia. Aside from these we find, also, a quantity of aqueous vapor.

To what variations are these various gases subject? The relative proportions of oxygen and nitrogen are, by weight, 77 of the former to 23 of the latter. It is found that this proportion is uniform. Although there is no chemical union between these two component elements, yet, so uniform is the operation of the law of diffusion of gases, that the atmosphere is found to be relatively the same in all localities, and at all altitudes as yet attained. This uniform proportional condition renders it impossible that causes of disease should exist in the elements of oxygen and nitrogen.

Carbonic acid should be considered as one of the normal ingredients of a healthy atmosphere. It is always present, and is highly necessary to vegetation. Its quantity is, however, so small, and its range of variation so limited, that we cannot assign to it any share in the causation of disease. It is found in the small proportion of from 3.7 to 6.2 measures in 10,000 measures of air. Ammonia, like carbonic acid, is inappreciable as a cause of disease. Only a trace of it is found at any time. It should, however, be stated, that the proportion of carbonic acid depends upon circumstances within the control of man, and that the calculations given are intended to represent the air in its natural condition. Its quantity depends upon two causes: 1st, the amount of it produced by man in respiration and in the consumption of fuel; and, also, by the respiration of animals; and 2d, upon the amount of it absorbed in the processes of vegetation. Thus, a thinly inhabited and heavily wooded country will have less carbonic acid than a great city, where there are not sufficient trees to absorb it; and which must depend on perflation by winds, for its disposal. Of course any excess of carbonic acid is hurtful, and it undoubtedly is one of the causes of disease in cities.

Causes analogous favor the production of ammonia. The decay of animal products, the vapor of cess-pools and sewers, increases materially the amount of free ammonia in the air of cities. Ammonia is fatal to animal life in a much less proportion than is carbonic acid.

We have, then, but one condition left, which, in its amount and range of variation, can justify investigation as a cause of disease. We find that humidity is a constant, but very variable component of the atmosphere. A perfectly dry atmosphere would soon be fatal to both animal and vegetable life. We find that the amount of variation in a cubic foot of vapor ranges, by weight, from 0.856 gr. to 17.009 grs., within the ordinary range of variation. Either of these extremes would be most injurious to health.

The amount of humidity depends very much upon the temperature of the air. Thus, in a frosty or freezing temperature we have the nearest approximation to dryness. All moisture existing in air is frozen and precipitated, leaving the air itself in a condition of approximate dryness. It may be considered as the normal rule, that, as we increase the temperature, we increase the humidity. The capacity of the atmosphere for moisture increases with its temperature. Thus the cubic foot of vapor which, at 0°, weighed less than a grain, will, for each additional degree of temperature, acquire an additional modicum, increasing with the temperature. Thus, a cubic foot of vapor at 1°, will weigh .036 gr. more than a cubic foot at zero. But we find a cubic foot at 60° weighs 0.176 gr. more than at 59°, while at 95° it will weigh 0.416 gr. more than at 94°.

This *power* of increasing capacity does not, however, indicate the *actual fact* of an increased humidity. An atmosphere at 95° may be relatively dryer than one at 60°. The real humidity depends upon the source of supply. If copious rains have fallen, and the surface of the earth is saturated with moisture, the actual humidity will be great in proportion to the height of the thermometer; though the relative humidity may not express a corresponding difference in value. As an illustration, I take two days from the record of the latter half of May. On the 17th, at 2 P. M., the thermometer stood at 76°. The relative humidity was 0.615; 1000 representing saturation. The actual weight of a cubic foot of vapor on that day was 6.031 grs., requiring for saturation an addition of 4.076 grs. On the 26th, during the fog which followed the rain in the morning, the thermometer stood, at 2 P. M., at 66°. The relative humidity was 0.787. The actual weight of a cubic foot of vapor on that day was 5.575 grs., requiring for saturation* only 1.872 grs.

* "Saturation is incorrect as a term. The condition of maximum density for a given temperature is what is intended by the term *saturation*.

It will be seen from this statement, that the relative humidity does by no means express the amount of humidity, but only its relation to the amount that the air is capable of containing at a given temperature.

The increasing capacity of air for vapor, at increasing temperatures, is doubtless a general law of nature; intended to ward off the deleterious effects of too great dryness, combined with heat, which would be fatal to vegetation. In the normal condition of the atmosphere there should therefore be a sufficient amount of vapor present, to preserve a certain relative proportion between the two. Whatever may be the actual amount of humidity, its proportion to the degree of saturation cannot vary to any great extent without affecting both animal life and vegetation.

And, consequently, we may argue, that a humidity above the mean, combined with a high temperature, will be injurious to health and vegetation, from an excess of actual moisture. This effect upon vegetation is manifested in the cereals, by the sudden appearance of "rust" in wheat, after a spell of warm, wet weather. In this disease of wheat, the plant is overcharged with juices, congestion occurs, followed by effusion and splitting, or rupture of the integument, and consequent destruction of the plant.

Its effect upon man is to be the subject of our inquiry.

To ascertain the relative humidity of the air, various instruments called hygrometers have been invented. That now most generally in use, as combining convenience, accuracy, and cheapness, is Mason's instrument, which consists of two thermometers upon the same frame, one of which is exposed to the ordinary vicissitudes of the atmosphere, while the bulb of the other is kept constantly wetted by capillary attraction. Should the atmosphere be saturated with moisture, of course both thermometers will register the same degree upon the scale. But, as we approach dryness, the temperature of evaporation will become less than the atmospheric temperature, and we shall have an increasing difference in the register of the two thermometers.

Upon this simple instrument, assisted by tables and problems, which have been compiled from experiment, we are enabled to ascertain, at any moment, the temperature of the air, of evaporation, and of the dew point; the number of grains weight in a cubic foot of vapor at that time, and the relative humidity.

I furnish, finally, to the Association, a record of the temperature and humidity of the atmosphere from May 17th to 31st, inclusive. In it, 1000 represents saturation, and 0, complete dryness—500 will approximately represent the mean, or normal humidity of a year.

| May, 1854. Day of Month. | Dry Bulb. | | Wet Bulb. | | Relative Humidity. | |
|---|---|---|---|---|---|---|
| | Upper Station. | Lower Station. | Upper Station. | Lower Station. | Upper Station. | Lower Station. |
| 17 | 76 | | 69 | | 615 | |
| 18 | 64 | | 59 | | 689 | |
| 19 | 65 | | 59 | | 641 | |
| 20 | 59 | | 54 | | 686 | |
| 21 | 58 | | 55 | | 790 | |
| 22 | 59 | | 55 | | 750 | |
| 23 | 63 | 63 | 56 | 58 | 601 | 689 |
| 24 | 68 | 68 | 62 | 62 | 654 | 655 |
| 25 | 68 | 67 | 64 | 63 | 743 | 765 |
| 26 | 66 | 68 | 62.5 | 63 | 787 | 698 |
| 27 | 70 | 68 | 62 | 62 | 558 | 654 |
| 28 | 72 | 70 | 64 | 64 | 560 | 656 |
| 29 | 72 | 70 | 65 | 65 | 598 | 697 |
| 30 | 70 | 68 | 59 | 62 | 448 | 642 |
| 31 | 66 | 64 | 62 | 60 | 762 | 761 |
| Average, . . . . . . . . . . . . | 67.06 | 67.33 | 60.46 | 62.11 | 639* | 690 |

* Calculated for last nine days only.

| | Upper Station. | Lower Station. |
|---|---|---|
| Greatest Heat on any day, (in last 9 days,) . . | 72° | 70° |
| Lowest " " " " . . . | 63° | 63° |
| Highest Humidity on any day, (in last 9 days,) . | .787 | .765 |
| Lowest " " " . . | .448 | .642 |
| Height of Stations above Lake Erie, . . . | 65 feet. | 20 feet. |
| " " " level of the Ocean, (about) | 606 feet. | 561 feet. |

*Memorandum of Levels in Main Street:*

Centre of Main and Ohio Street, is above the Lake . . . 4 feet.
Centre of Main and Eagle Street, " " . . . 45 feet.
Centre of Main and High Street, " " . . . 88 feet.

It is seen from this comparative view, that the humidity is greater at the lower station, in Merchants' Exchange, than at the upper, at 302 Main Street. This is due, either to altitude, or the proximity of the lower station to the docks. The establishment of a station upon the level of High Street, will test the relative influence of altitude, as distinguished from local causes.

